# The Modern Medical Journal

**Published:** 1910-10-01

**Authors:** 


### The Hospital

A Journal of

The Medical Sciences and Hospital Administration.

New Series. No. 190, Vol. VIII. [No. 1262, Vol. XLIX.] SATURDAY, OCTOBER 1, 1910.

### The Modern Medical Journal.

On occasions it is allowable for a journal to talk about itself, and though custom has decreed that the self-explanatory note should only intrude at more or less fixed periods, it has nevertheless allowed some latitude as to the interpretation of this rule. Justifiably the personal string may be twanged when a paper starts its career or at certain epochs of its existence, as when it enters upon a new phase or annually commences a new edition. The present is such an occasion, for with this number The Hospital commences its forty-ninth year and the eighth edition of its rejuvenated and amplified reformation, which gives it some right to style itself the Modern Medical Journal.

Years ago we decided that this paper should fill the want, at that time very evident, felt by the practitioner and the institutional worker for a journal that could be a medium of information on the many important points that interest both. The nursing section of the paper was therefore separated into a special journal, and The Hospital given over solely to the interests of the general practitioner and the hospital worker. The innovation, we are glad to state, has been well received, and during the past few years we have had ample encouragement to continue the programme outlined in the first number of the new series—encouragement for which we are cordially grateful to those who have given us their support and sympathy. In the new edition which starts with this issue, we trust, by adhering to that outlined programme, by attending strictly to the dual interest for which this journal exists, and by increasing and extending our scope of usefulness, to merit yet further support. We desire to make The Hospital a practically useful practitioner's weekly, and to succeed in that endeavour we need the help of everyone who is interested in the existence of such a journal. Medical science and institutional work progress so rapidly in these days that there is need for interpretation and interpreters. The mass of available literature in all branches of these two subjects is too vast and unwieldy to permit the busy professional man to follow it for himself. At the same time there is need for a magazine that shall be not merely scientific, but that shall also deal with the many important problems of semi-scientific nature that confront the professional man to-day. Gradually the interests of hospital worker and doctor are beginning to overlap, and now more than ever it behoves both to be acquainted with the salient features of each other's special field of labour. The modern medical journal must concern itself with both, and it must not lose sight of the fact that there are other fields that encroach upon its special scientific domain—fields not only of work but of relaxation, problems not merely of to-day but of to-morrow.

The past numbers of the new series of The Hospital are sufficient proof of the fact that we have not overlooked these necessities. Our aim is to appeal to the two classes whose interests are so closely akin, and as in the past we will continue to cater for both. The medical section of the journal will deal with subjects that are of special importance to the profession, always with the proviso that our first consideration is the general practitioner, not the specialist, nor yet the student. Week by week we shall review, as heretofore, important questions of the moment in the form of leading articles or annotations, and in the six columns of Medical Opinion and Movement provide our readers with a running commentary on professional work and methods in the shape of excerpts from all the important medical papers, both English and foreign. The series of clinics, each by an authority in his special branch and each chosen solely for its practical value, will be continued, and will be followed by special articles on outstanding questions of the day, new methods, theories, and discoveries. The departmental articles, covering the whole field of practical medicine, and edited by experts who have specially identified themselves with each separate division of work, will also be continued, and in addition, in our news columns and in the special sections dealing with medical societies, post-graduate and parliamentary work, full attention will be paid to current changes and developments. The series of articles contributed by general practitioners and on the practitioner's relaxations and hobbies will be extended, and made more catholic and, as we trust, more generally useful to the practitioner. In the Institutional section we shall deal, again as heretofore, with every subject that should interest those concerned in the work and administration of our charitable institutions. In the Week's Work columns we shall pass in review the latest developments of importance in the hospital world. Special articles dealing with subjects of institutional interest, hospital construction, plans, and sketches, interviews with prominent workers in the field, epitomes and abstracts of foreign progress all along the line, news and notes from institutional sources, and the many other special features of this section will be continued; and here, as in the medical portion of the paper, we hope to prove plain commentators and live reporters, not content with holding glimmering rushlights to the sun, but willing to exploit the whole range of the very wide acreage of information around us.

These are high hopes and lofty ambitions, and they cannot be realised except with the co-operation and support of all our readers. No journal can stand by itself, singly, especially one that professes to burden itself with the interests of particular classes. We want, therefore, a quick and active interest in our programme; we want contributions, help, above all, criticism. Let us know in what we err and how, so as to enable us to improve and amend. To the practitioner we will take leave to say that his own immediate experiences in practice may prove helpful to brother practitioners; let us have them. To the institutional worker, secretary, committee-man, or voluntary helper, that his work, no matter in how limited a sphere, may hold valuable instruction to other workers; send us the record, even of incidents. There are, after all, so many things that have to do with Iphicles' oxen! And it is sometimes hard to collect all that relates to the herd. We are grateful for support rendered us in the past, and we look for similar support, with confidence, in the future. For we are satisfied that The Hospital fills a want, and we are assured that it can claim with honesty, if its readers take an interest in its development, to be truly the practitioner's modern medical journal.

